# Clinical parameters in different stages, zones, and remission/progression statuses of retinopathy of prematurity

**DOI:** 10.22088/cjim.13.2.10

**Published:** 2022

**Authors:** Seyed Ahmad Rasoulinejad, Faezeh Maroufi, Ahad Alizadeh

**Affiliations:** 1 Department of Ophthalmology, Ayatollah Rouhani Hospital, Babol University of Medical Sciences, Babol, Iran.; 2 Department of Medical Laboratory Sciences, Faculty of Allied Medicine, Qazvin University of Medical Sciences, Qazvin, Iran; 3 Metabolic Diseases Research Center, Qazvin University of Medical Sciences, Qazvin, Iran

**Keywords:** Retinopathy of prematurity, Prognosis, Laboratory parameters, Hematologic parameters, Biochemical parameters.

## Abstract

**Background:**

Retinopathy of prematurity (ROP) is a vasoproliferative retinal disease in premature infants that causes lifetime visual impairment and blindness in the early ages. In this study, we investigated the differences in the values of clinical laboratory parameters between different ROP and its remission/progression statuses regarding stages and zones.

**Methods:**

This historical cohort study includes 828 infants divided into two groups after the first examination containing ROP infants and controls. The biochemical and hematological parameters of the two groups have been collected from the patient’s history.

**Results:**

In infants with ROP, the hematopoiesis-related parameters, including the mean level of hemoglobin, total bilirubin, potassium, calcium were significantly less than controls (P=0.039, P=0.001, P=0.001, and P=0.046, respectively). The percentages of reticulocyte and the levels of BUN in ROP patients were significantly higher than in normal infants (P=0.015 and p <0.001, respectively). Moreover, the levels of hemoglobin and BUN were significantly different in the different zones of ROP (P=0.017 and P=0.001, respectively). Also, higher hemoglobin levels, total bilirubin, and CRP were observed in the reduced stages of ROP (P=0.041, P=0.045, and P=0.039, respectively).

**Conclusion:**

Laboratory parameters are different in different stages, zones and remission/ progression ROP infants.

Retinopathy of prematurity (ROP) is a vasoproliferative retinal disease in premature infants that causes lifetime visual impairment and blindness in the early ages (1-4), which imposes an enormous economic and psychological burden on the government and society. ROP is characterized by abnormal intravitreal neovascularization in the premature retina (5-7). ROP severity has been divided into five stages: starting with the primary phase and anticipating advanced ROP that comes with hemorrhage, fibrovascular alterations, vitreoretinal traction, and retinal detachment (8, 9). Moreover, three zones of the retina are involved in ROP, indicating the rate of ROP involvement, including Zone I: The area, defined by a circle centered on the optic nerve; Zone II: The area, extends centrifugally from the edge of Zone I; Zone III: The residual temporal crescent of retina anterior to Zone II. Many studies have proven the relationship between the pathogenesis of ROP and its risk factors such as low birth weight, short gestational age, hypoxia, and other factors (10-13). Previous studies have demonstrated that the amount of hemoglobin and count of leukocytes and platelets can influence ROP rising and development; thus, they can be considered prognostic factors (14-19). 

To prevent the development of ROP and on-time treatment of affected infants, identifying high-risk infants is essential through predictive factors. In this study, we have analyzed the value of some biochemical and hematological parameters of the blood of premature infants including the number of white blood cells (WBC), platelets (Plt), hemoglobin (Hb), reticulocytes, total and direct bilirubin (Bili T, Bili D), blood urea nitrogen (BUN), creatinine (Cr), sodium (Na), potassium (K), calcium, blood sugar, C-reactive protein (CRP) and pH to investigate the differences between different ROP and its remission/ progression statuses regarding stages and zones.

## Methods

This historical cohort study was administered in the Ophthalmology Center of Ayatollah Rouhani Hospital in Babol, which affiliates with Babol University of Medical Sciences (Babol, Iran) from 2010 to 2020. It included 828 infants (<37 weeks of gestation with a birth weight <2500 g). Institutional ethics committee approval was obtained from the local ethics committee (IR.MUBABOL.REC.1399.373). Initial examinations were performed four weeks after birth. For this aim, one hour after administering 2.5% phenylephrine and 0.5% tropicamide, the funduscopic examinations were implemented using a binocular indirect ophthalmoscope, 28D lens, scleral depressor, and pediatric speculum. If there was an ROP, the second examination was conducted two weeks after the first examination. The treatment protocol, including anti-vascular endothelial growth factor injection, was conducted according to the international classification of retinopathy of prematurity (ICROP) criteria for patients in stage 3 or more. The infants were separated into two groups, including the infants with no signs of ROP as the control group and infants with different stages of ROP as the case group. Of course, there were only stages 1, 2, and 3 of ROP in patients, and none of the infants had stages 4 or 5. 

Blood samples were obtained from the infant’s veins; after that, biochemical and hematological parameters of the two groups were examined by an automated system. These parameters and their reference ranges at first month after birth include white blood cell count (WBC, 6.0-18.0×10^9 ^cell/L), hemoglobin (Hb, 13.4-19.9 g/dL), platelet count (Plt, 180-400 × 10ꝰ/L), reticulocyte percentage (2.5-6.5% of RBCs), bilirubin total (Bili T, 0.1-12.0 mg/dL), bilirubin direct (Bili D, 0.1-2 mg/dL), blood urea nitrogen (BUN, 3-12 mg/dL), creatinine (Cr, 5-18 mg/dL), sodium (Na, 134-144 mEq/L), potassium (K, 3.9-5.9 mEq/L), calcium (Ca, 5.9-11.0 mg/dL), blood Sugar (100-200 mg/dL), C-reactive protein (CRP, <1.0 mg/L), pH (7.35-7.45). Statistical analysis was performed using the SPSS 21.0 software. Quantitative variables were reported with mean ±standard deviation (SD). Chi-square and independent t-test evaluated univariate comparisons of risk factors between the groups. The level of significance was taken to be p<0.05 for all statistical tests.

## Results

**Patient characteristics: **A total of 828 premature neonates were included in this study. Between participants, 525 infants were normal individuals and 303 infants were identified with ROP, and 124 cases were treated. In the case group, 135 were females and 163 were males (5 missed data), and in the control group, 283 were females, and 242 were males. Laboratory parameters were obtained from all of them, and their data were compared for different items. The patients with ROP had different stages; 106 of them had stage 1, 115 of them had stage 2, and 76 of them had stage 3 of ROP (4 missed data). Moreover, 62 of the infants had ROP involvement in Zone 1, 156 in Zone 2, and 77 in Zone 3 (8 missed data). In the second examination, ROP had improved in 158 patients (157 fully treated and 1 case with reduction in stage). 

**The values of the measured** **laboratory parameters in ROP and non-ROP infants: **As mentioned above, in the first examination, two groups were separated: the control group consists of infants without ROP, and the case group includes infants with different ROP stages. The mean amount of hemoglobin in normal infants was 14.44±3.31 g/dl, but in infants with ROP, the mean amount of Hb was 13.82±3.30 g/dl (P=0.039). In the following of the reduction of Hb in infants with ROP, their RBC production increased consequently; therefore, the percentage of reticulocytes in ROP patients was significantly higher than normal infants (3.97±2.53 x10^6^cell/µl and 3.09±2.08 x10^6^cell/µl, respectively, P=0.015). Moreover, there was no significant difference between the count of WBC and Plt in normal infants and infants with ROP (P=0.110 and P=0.093, respectively). Also, obvious differences in biochemical factors were observed between infants with ROP and infants without ROP. The mean level of total bilirubin in infants without ROP was 8.61±3.83 mg/dL, and in ROP patients were significantly lower (7.30±3.65 mg/dL, P=0.001). However, there was no significant relevancy between direct bilirubin and ROP (P=0.059). The BUN levels in infants with ROP were 17.34±10.54 mg/dL and higher than the normal range, but the BUN level in the control group was 12.81±7.34 mg/dL (p<0.001). Through the serum electrolytes, only potassium and calcium may play a significant role in ROP (P=0.001 and P=0.046, respectively). There was no significant difference between sodium levels in infants with ROP and normal individuals (P=0.557). The mean level of potassium in normal infants was 4.94±0.82 mEq/L and significantly higher than potassium levels in infants with ROP (4.63 ± 0.86 mEq/L). Similarly, the mean calcium level in the control group was 9.02± 1.53 mg/dL and significantly higher than the case group with the mean level of 8.72 ± 1.11mg/dL. In the following, the blood pH did not significantly associate with ROP (P =0.787). The inflammatory index of CRP did not show a significant increase in infants with ROP (P=0.495) (table 1).

**Table 1:** The values of laboratory parameters in the case and control group

**Parameters**	**Control** **Control**	**Case**	**P-value**
WBC (cell/ µl) 0	12609.24 ± 19368.05	10558.37 ± 10893.28	0.110
Hb (g/dL)	14.44 ± 3.31	13.82 ± 3.30	0.039
Plt (cell/ µl)	275784.80 ± 130346.17	256491.72 ± 121153.73	0.093
Reticulocyte (% of RBC)	3.09 ± 2.08	3.97 ± 2.53	0.015
Bili T (mg/dL)	8.61 ± 3.83	7.30 ± 3.65	0.001
Bili D (mg/dL)	0.41 ± 0.56	0.34 ± 0.14	0.059
BUN (mg/dL)	12.81 ± 7.34	17.34 ± 10.54	<0.001
Cr (mg/dL)	0.58 ± 0.35	0.71 ± 0.87	0.065
Na (mEq/L)	136.91 ± 4.99	136.30 ± 12.22	0.557
K (mEq/L)	4.94 ± 0.82	4.63 ± 0.86	0.001
blood Sugar (mg/dL)	70.30 ± 36.85	85.49 ± 57.20	0.095
Ca (mEq/L)	9.02 ± 1.53	8.72 ± 1.11	0.046
CRP (mg/L)	4.68 ± 20.38	3.69 ± 7.28	0.495
pH	7.33 ± 0.09	7.33 ± 0.10	0.787

**The difference in laboratory parameters in different stages of ROP: **Regarding the analysis of the values of each parameter in stages 1, 2, and 3 of ROP, no significant relationship was found between the level of parameters and their changes in different stages (table 2).

**The difference in laboratory parameters in different zones of ROP: **The comparison of laboratory parameters in different zones of ROP showed significant differences in hemoglobin and BUN levels at different zones of ROP (P =0.017 and P=0.001, respectively). The level of Hb was higher in Zone 3 compared to other zones, and the mean levels of BUN in Zone 1 of ROP were higher than the normal range in infants and showed a significant increase compared to different zones (table 3). Other parameters did not significantly correlate with the zones involved in ROP.

**The difference in laboratory parameters in different statuses of remission/progression of ROP: **Patients diagnosed with advanced ROP in the first examination were treated and re-examined after two weeks. Table 4 demonstrates the difference between the first and second examination of ROP, which determines the improvement of disease. Our results have shown that in cured patients, the mean count of WBCs was significantly less than in un-cured infants (P=0.006). Also, the mean levels of CRP as inflammatory index in cured infants were significantly less than in un-cured patients (P=0.011) and WBCs. This evidence indicates that in patients with higher immune levels, the response to treatment and disease improvement are weaker. The serum level of potassium in patients involved with ROP was 4.91±0.80 mEq/L and in cured infants was 4.61±0.85 mEq/l; therefore, the increased potassium levels in un-cured patients were significantly associated with a less remission and weaker response to treatment (P=0.008). Also, the total bilirubin has shown higher levels in un-cured infants, which is significantly associated with a lack of complete remission in patients (P=0.030). Other factors have shown no significant relation with ROP remission (table 4).

**The** **difference of means of** **laboratory parameters in different progression statuses of the stage of ROP: **The purpose of this section is to analyze the differences between the stage of ROP in the first and second examination and evaluate the progression or improvement of ROP in un-cured infants. The reduction of the stages in the second examination indicates the recovery of the ROP. Our results revealed that the hemoglobin, total bilirubin, and CRP have a significant relationship with changing the stage of ROP (P=0.041, P =0.045, and P=0.039, respectively) (table 5). In patients with a reduced stage, the mean hemoglobin levels were 16.00±1.90 mg/dl, higher than other conditions. The stable patients in terms of ROP stages (no change in status of the stage between two exams) had mean hemoglobin levels of 12.98 ± 3.61 mg/dl. In addition, the mean hemoglobin level in patients with progressed ROP (increasing in stage) was 14.62±3.52 mg/dl. As well as the hemoglobin levels, the mean total bilirubin in patients with a reduced stage was 9.55±1.77 mg/dl.

**The** **difference of means of** **laboratory parameters in different progression statuses of the zone of ROP: **Similar to the previous section, the differences between the zone of ROP in the first and the second examinations indicate the progression or improvement of ROP in involved infants. Moreover, the increase of zone in the second examination shows the remission of the ROP. With regard to our results, there was not any significant relationship between laboratory parameters and changing of the zones of ROP (table 6). 

**Table 2:** The values of laboratory parameters in different stages of ROP

**Parameters**	**Patients in Stage 1**	**Patients in Stage 2**	**Patients in Stage 3**	**P value**
WBC (cell/ µl) 0	10985.17 ± 12224.81	8983.07 ± 6822.11	12223.81 ± 13284.85	0.251
Hb (g/dL)	13.94 ± 3.46	14.17 ± 3.19	13.17 ± 3.15	0.325
Plt (cell/ µl)	259820.00± 119571.42	256194.66± 125991.62	250162.79± 122801.22	0.726
Reticulocyte (% of RBC)	3.34 ± 1.83	4.58 ± 2.32	4.50 ± 4.18	0.122
Bili T (mg/dL)	7.30 ± 4.21	7.31 ± 3.18	7.28 ± 2.82	0.781
Bili D (mg/dL)	0.32 ± 0.13	0.36 ± 0.15	0.33 ± 0.15	0.478
BUN (mg/dL)	16.77 ± 11.11	16.95 ± 8.51	19.72 ± 11.88	0.307
Cr (mg/dL)	0.61 ± 0.20	0.96 ± 1.53	0.60 ± 0.19	0.437
Na (mEq/L)	138.13 ± 5.34	133.06 ± 20.42	136.60 ± 6.25	0.093
K (mEq/L)	4.71 ± 0.77	4.55 ± 0.95	4.57 ± 0.95	0.699
blood Sugar (mg/dL)	74.47 ± 40.27	71.44 ± 49.40	116.14 ± 73.03	0.066
Ca (mEq/L)	8.77 ± 1.12	8.61 ± 0.87	8.87 ± 1.38	0.425
CRP (mg/L)	4.43 ± 8.50	3.63 ± 7.13	2.09 ± 3.21	0.329
pH	7.35 ± 0.21	7.33 ± 0.12	7.35 ± 0.08	0.312

**Table 3:** The values of laboratory parameters in different zones of ROP

**Parameters**	**Zone 1**	**Zone 2**	**Zone 3**	**P value**
WBC (cell/ µl) 0	10647.39 ± 11541.14	9641.02 ± 7906.21	12173.41 ± 14827.92	0.390
Hb (g/dL)	13.87 ± 3.02	13.29 ± 3.37	14.86 ± 3.20	0.017
Plt (cell/ µl)	252351.35± 121992.99	253485.55± 125551.06	263050.00± 119745.25	0.791
Reticulocyte (% of RBC)	4.32 ± 4.08	4.26 ± 2.24	3.51 ± 2.19	0.420
Bili T (mg/dL)	7.85 ± 3.22	6.59 ± 3.00	8.17 ± 4.68	0.072
Bili D (mg/dL)	0.36 ± 0.15	0.34 ± 0.16	0.32 ± 0.10	0.642
BUN (mg/dL)	23.01 ± 12.82	17.53 ± 10.37	13.77 ± 7.35	0.001
Cr (mg/dL)	0.69 ± 0.27	0.80 ± 1.21	0.60 ± 0.15	0.563
Na (mEq/L)	136.64 ± 5.98	135.25 ± 16.47	137.66 ± 5.42	0.820
K (mEq/L)	4.75 ± 0.73	4.60 ± 0.94	4.59 ± 0.85	0.722
blood Sugar (mg/dL)	105.09 ± 34.53	85.96 ± 71.27	70.42 ± 42.81	0.111
Ca (mEq/L)	8.64 ± 1.31	8.77 ± 1.05	8.79 ± 1.09	0.801
CRP (mg/L)	2.19 ± 4.09	4.30 ± 7.99	3.79 ± 7.74	0.197
pH	7.33 ± 0.08	7.34 ± 0.11	7.36 ± 0.28	0.423

**Table 4:** The difference of means of laboratory parameters with the improvement of ROP

**Parameters**	**level**	**no change in ROP status**	**ROP remission**	**P-value**
WBC (cell/ µl) 0	NA	12338.94 ± 18226.66	9982.42 ± 10281.81	0.006
Hb (g/dL)	NA	14.30 ± 3.37	13.71 ± 3.17	0.061
Plt (cell/ µl)	NA	267771.68 ± 130364.00	272033.33 ± 119755.19	0.614
Reticulocyte (% of RBC)	NA	3.26 ± 2.13	3.94 ± 2.71	0.138
Bili T (mg/dL)	NA	8.35 ± 3.76	7.55 ± 4.12	0.030
Bili D (mg/dL)	NA	0.40 ± 0.51	0.34 ± 0.15	0.442
BUN (mg/dL)	NA	14.20 ± 8.88	15.66 ± 9.62	0.111
Cr (mg/dL)	NA	0.61 ± 0.51	0.70 ± 0.91	0.093
Na (mEq/L)	NA	136.72 ± 5.10	136.10 ± 15.36	0.227
K (mEq/L)	NA	4.91 ± 0.80	4.61 ± 0.85	0.008
blood Sugar (mg/dL)	NA	71.38 ± 36.97	73.92 ± 44.47	0.696
Ca (mEq/L)	NA	8.91 ± 1.49	8.84 ± 1.06	0.947
CRP (mg/L)	NA	4.84 ± 18.91	2.91 ± 6.08	0.011
pH	NA	7.33 ± 0.10	7.35 ± 0.19	0.871

**Table 5:** The difference of means of laboratory parameters with the changing the stage of ROP

**Parameters**	**decreasing in stage**	**no change in stage**	**increasing in stage**	**P-value**
WBC (cell/ µl) 0	16662.50 ± 23224.74	10658.31 ± 9556.31	9343.79 ± 5701.15	0.761
Hb (g/dL)	16.00 ± 1.90	12.98 ± 3.61	14.62 ± 3.52	0.041
Plt (cell/ µl)	205125.0± 120971.59	244305.8± 135207.57	215812.5± 98890.66	0.701
Reticulocyte (% of RBC)	4.83 ± 1.27	3.79 ± 1.94	5.02 ± 3.36	0.494
Bili T (mg/dL)	9.55 ± 1.77	6.55 ± 2.61	6.72 ± 3.76	0.045
Bili D (mg/dL)	0.32 ± 0.04	0.35 ± 0.12	0.34 ± 0.13	0.882
BUN (mg/dL)	25.56 ± 13.67	18.21 ± 9.46	20.56 ± 15.32	0.332
Cr (mg/dL)	0.56 ± 0.21	0.62 ± 0.20	1.10 ± 1.71	0.361
Na (mEq/L)	136.62 ± 6.57	136.03 ± 4.83	135.64 ± 6.64	0.994
K (mEq/L)	4.97 ± 0.87	4.70 ± 0.64	4.89 ± 0.84	0.663
blood Sugar (mg/dL)	8.27 ± 1.63	8.40 ± 1.22	8.62 ± 0.65	0.855
Ca (mEq/L)	4.00 ± 5.92	4.20 ± 8.40	10.18 ± 12.66	0.039
CRP (mg/L)	7.34 ± 0.06	7.34 ± 0.13	7.31 ± 0.10	0.796

**Table 6:** The difference of means of laboratory parameters with the changing the zone of ROP

**Parameters**	**Decreasing in zone**	**Decreasing No change in zone**	**with Decreasing Increase in zone**	**P-value**
WBC (cell/ µl) 0	7566.67 ± 2325.22	11374.11 ± 9655.97	11458.86 ± 16691.32	0.214
Hb (g/dL)	13.75 ± 5.29	13.32 ± 3.50	14.42 ± 3.23	0.538
Plt (cell/ µl)	231666.67± 160128.28	224458.02± 118744.48	252562.50± 143634.24	0.926
Reticulocyte (% of RBC)	5.92 ± 2.59	6.68 ± 3.21	7.65 ± 2.68	0.348
Bili T (mg/dL)	0.32 ± 0.10	0.37 ± 0.13	0.30 ± 0.06	0.401
Bili D (mg/dL)	15.96 ± 13.30	20.93 ± 12.17	19.74 ± 12.03	0.388
BUN (mg/dL)	1.92 ± 2.85	0.60 ± 0.20	0.65 ± 0.22	0.427
Cr (mg/dL)	134.50 ± 3.51	136.80 ± 4.85	134.82 ± 6.79	0.381
Na (mEq/L)	4.75 ± 0.77	4.71 ± 0.76	4.89 ± 0.70	0.750
K (mEq/L)	6.55 ± 1.91	8.80 ± 0.88	8.03 ± 1.27	0.066
blood Sugar (mg/dL)	11.83 ± 18.32	5.19 ± 8.48	4.31 ± 5.99	0.628
Ca (mEq/L)	7.30 ± 0.06	7.34 ± 0.15	7.33 ± 0.04	0.786

## Discussion

Retinopathy of prematurity (ROP) is a vascular retinopathy disease in premature infants that causes vision loss. ROP is characterized by abnormal intravitreal neovascularization in the retina due to arrest in normal retinal vascular development, resulting in ischemia and retinal hypoxia. Many factors associated with ROP development include low birth weight, short gestational age, inflammation, mutations, supplemental oxygen therapy, and its relationship with hyperoxia dominate. An increase of vascular endothelial growth factor (VEGF) and its production and secretion is identified as a primary risk factor of ROP in the retina regeneration period. 

Previous studies have demonstrated that the laboratory parameters like hematological and biochemical factors can be influenced in ROP rising and progression, and they can be considered prognostic factors. In this study, we have investigated the association of biochemical and hematological parameters with ROP prognosis in premature infants. 

According to the results, the reduction of hemoglobin and increase in reticulocytes show that mild anemia was present in infants with ROP; thus, mild anemia is a risk factor for ROP. It was related to oxygen therapy and retinal vasculature autoregulation, and subsequent ischemia (20). In hyperoxia, the response of retinal blood flow (RBF) and choroidal blood flow (ChBF) of the newborn cannot be maintained constant; as a consequence, a huge amount of toxic oxygen delivery into the retina can upset the autoregulatory response (21). Accordingly, reducing oxygen supplementation has been repeatedly described as an effective factor in declining ROP rate (22, 23).

The lower levels of total bilirubin, potassium, and calcium have presented in ROP cases compared with non-ROP infants, suggesting that the high levels of bilirubin, potassium, and calcium can be considered a protective factor against ROP rising and development. Some studies have shown that bilirubin has an antioxidant role in vitro and can protect against oxidative stress (24), so studies tend to manifest the association of serum bilirubin level with ROP. On the other hand, the photoreceptor calcium channels have a role in ion flux to prevent the oxidative stress; thus lower level of calcium has an effective role in the development of the lesions of ROP (25). Also, the higher levels of BUN were observed in ROP cases. Moreover, the levels of hemoglobin and BUN significantly related with the zone of ROP, in which the level of Hb was higher in Zone 3; the mean level of BUN in Zone 1 of ROP was higher than in other zones. On the other hand, the higher levels of hemoglobin, total bilirubin, and CRP, as a prognostic factor, can predict the stage reduction of ROP that is associated with the improvement of ROP. In addition, the higher levels of WBCs, CRP, potassium, and total bilirubin can be considered a prognostic factor to predict the development of ROP and response to treatment. Therefore, the response to treatment is weaker in patients with higher immune levels related to higher inflammation status. 

Other similar studies confirm our results. Heyman et al. suggested that a high bilirubin level may have a protective role as an antioxidant on ROP progression (26). Also, Akkawi et al. found that the high bilirubin levels are a protective factor against ROP development. Also, they suggested the low hemoglobin level can be considered a risk factor for developing ROP in infants (27). Similarly, in a Chinese study, anemia was identified as an independent risk factor of ROP (28). Ünsal et al. demonstrated that hemoglobin and related parameters values in infants with ROP were significantly lower than the normal infants. Leukocyte count was higher in the ROP group (29). Our results suggest that the high levels of leukocytes can be considered a prognostic factor for ROP development. Kurtul et al. found that inflammation in ROP leads to an increase of WBC and neutrophil counts; thus, the neutrophil-lymphocyte ratio can be considered a predictive factor for ROP (15). 

Our results did not show any differences in platelet count between ROP cases and controls; in contrast, several studies have indicated that thrombocytopenia can be considered a prognostic factor for ROP (14, 18). Due to these outcomes, laboratory parameters can predict the prognosis of ROP and the risk of anticipated severe ROP; also they can be used as a simple screening test to predict the ROP (17, 30) that leads to on-time detection of ROP and early onset of treatment causing to prevent the progression of ROP into severe stages and blindness and can improve the life of infants.

Of course, our study had some defects, including its retrospective nature, failure to check further basic parameters like HbF and parameters of cord blood, lack of useful advanced methods such as PCR, chemiluminescence, or electrophoresis checking more precisely, and short follow-up. Despite these limitations, our results are promising for using the laboratory parameters as a simple screening tool to predict the ROP arising and prognosis. Future studies will identify other factors for predicting ROP prognosis and could contain inflammatory mediators, hemoglobin or leukocytes subgroups, specific antigens, mutations or polymorphisms, and underlying disease. In conclusion, Some laboratory parameters can effectively predict the prognosis of ROP. The lower levels of total bilirubin, potassium, calcium, and higher levels of BUN were presented in ROP cases compared to control infants. Moreover, the levels of hemoglobin and BUN are significantly related to the zone of ROP. In contrast, the higher levels of hemoglobin, total bilirubin, and CRP can predict the stage reduction associated with the improvement of ROP. In addition, the higher levels of WBCs, CRP, potassium, and total bilirubin can be considered a potent prognostic factor to predict the development of ROP and response to treatment. Due to these outcomes, laboratory parameters can be used as a simple screening test to predict ROP that leads to on-time detection of ROP and early onset of treatment causing to prevent the progression of ROP into severe stages and blindness. More studies are needed to find the correlations, regressions, precisions, and accuracies. 
